# Flood damage inspection and risk indexing data for an inventory of bridges in Central Greece

**DOI:** 10.1016/j.dib.2023.109062

**Published:** 2023-03-16

**Authors:** Marianna Loli, George Kefalas, Stavros Dafis, Stergios Aristoteles Mitoulis, Franziska Schmidt

**Affiliations:** a Grid Engineers, Athens, Greece; b International Hellenic University, Dept. of Forest and Natural Environment, Greece; c National Observatory of Athens, Institute of Environmental Research and Sustainable Development, Greece; Data4Risk, Paris, France; d University of Birmingham, UK; e Université Gustave Eiffel, France

**Keywords:** Floods, Extreme weather, Flood adaptation, Case study, Field data, Network, Risk analysis

## Abstract

This dataset is related to the research paper entitled “Bridge-specific flood risk assessment of transport networks using GIS and remotely sensed data” published in the Science of the Total Environment. It provides the information necessary for the reproduction of the case study that was used for the demonstration and validation of the proposed risk assessment framework. The latter integrates indicators for the assessment of hydraulic hazards and bridge vulnerability with a simple and operationally flexible protocol for the interpretation of bridge damage consequences on the serviceability of the transport network and on the affected socio-economic environment. The dataset encompasses (i) inventory data for the 117 bridges of the Karditsa Prefecture, in Central Greece, which were affected by a historic flood that followed the Mediterranean Hurricane (Medicane) Ianos, in September 2020; (ii) results of the risk assessment analysis, including the geospatial distribution of hazard, vulnerability, bridge damage, and associated consequences for the area's transport network; (iii) an extensive damage inspection record, compiled shortly after the Medicane, involving a sample of 16 (out of the 117) bridges of varying characteristics and damage levels, ranging from minimal damage to complete failure, which was used as a reference for validation of the proposed framework. The dataset is complemented by photos of the inspected bridges which facilitate the understanding of the observed bridge damage patterns. This information is intended to provide insights into the response of riverine bridges to severe floods and a thorough base for comparison and validation of flood hazard and risk mapping tools, potentially useful for engineers, asset managers, network operators and stakeholders involved in decision-making for climate adaptation of the road sector.

**Specifications Table**

**Table:** 

Subject	Engineering; Civil and Structural Engineering
Specific subject area	Management of natural hazard risk for infrastructure; Climate adaptation of transportation networks.
Type of data	Table (in one .xlsx document) Image (.jpg)
How the data were acquired	Damage inspection data were acquired through field reconnaissance visits to the locations of bridges affected by the flood that followed Medicane Ianos. The field visits, when the photos were captured, took place 2 weeks after the Medicane, i.e. 3-4 October 2020. In addition to traditional field reconnaissance and mapping methods, the authors employed UAVs for aerial mapping of damaged structures [6] and in-situ testing for the characterization of the concrete material in damaged structures [5]. A bridge inventory of all the bridges that exist in the County of Karditsa (117 structures), in Central Greece, was composed through the translation and processing of survey reports published by the Ministry of Environment and Energy of Greece: https://floods.ypeka.gr/index.php?option=com_content&view=article&id=1094&Itemid=799 Flood hazard, bridge vulnerability, potential consequences for the transport network, and associated risks are evaluated and listed for each bridge. They are outputs of the bridge-specific flood risk analysis framework presented in Loli et al., 2022.
Data format	Raw Analyzed
Description of data collection	The data collection process focused on riverine bridges existing in the County of Karditsa in Central Greece. The inventory data involves a total of 117 bridges. It documents the basic geometric properties of the bridge structures retrieved from reports (.pdf files) of surveys conducted by the Ministry of Environment and Energy of Greece. Documentation of damages was carried out amid travel restrictions and lockdowns due to the COVID-19 pandemic. Therefore, field inspections were carried out by groups of 2 – 3 persons and relied on image capturing and post-processing, to minimize social interactions and associated health risks. The field reconnaissance expedition was completed in two parts, from 3 – 4 October 2020 (15 days after the Medicane), and from 14 – 15 October 2023. Both ground and aerial images were captured, as detailed in the following. Measurements of key structural dimensions were carried out by use of LiDAR-equipped smartphones. Concrete material characterization was based on the extraction of cores from damaged bridge components. These were afterwards tested in compression in a local laboratory (see more information in [5]).
Data source location	City/Town/Region: County of Karditsa, Central Greece
Data accessibility	Repository name: Mendeley Data Data identification number: 10.17632/xsng4r5yhk.1 URL: https://data.mendeley.com/datasets/xsng4r5yhk
Related research article	Loli M, Kefalas G, Dafis S, Mitoulis SA, Schmidt F (2022) Bridge-specific flood risk assessment of transport networks using GIS and remotely sensed data. Vol. 850, 157976. https://doi.org/10.1016/j.scitotenv.2022.157976.

## Value of the Data

This dataset comprises two components, namely i) a thorough record of bridge damages in a historic, major flood, and ii) the results of a validated regional-scale assessment of flood risk in the area that was affected by the flood. Both can be useful for stakeholders involved in the management of climate risks for transport networks and in the design and planning of interventions for disaster risk reduction and climate adaptation. Such may be: government agencies, responsible for planning, designing, constructing, and maintaining transportation infrastructure; emergency services, such as police and fire brigade, interested in maintaining the functionality of critical road links after a disaster; transport operators responsible for safeguarding the day-to-day functionality and the resilience of their networks; as well as engineers and researchers involved in developing new methods for the analysis of climate risks and decision-making tools for planning of interventions. More specifically:

• This dataset facilitates the identification of patterns and trends of bridge damages in major floods, contributing to the understanding of the parameters that control their susceptibility to flooding, and the identification of design and construction practices that may lead to the dramatic amplification of hydraulic actions and failure of riverine bridges.
• This dataset is the first of its kind in that it documents the results of a large-scale application of the recently proposed French guidelines for the assessment of scour risk for bridges (CEREMA, 2019), considering the entire road network in a region with a total area of 2640 km^2^.
• It is also the result of the very first in the literature application of the Multi-Criteria-Decision-Making (MCDM) method for indexing flood velocity, a parameter that appears critical for the realistic evaluation of hazards due to flash flooding or river flooding in steep terrains (e.g., mountainous regions).
• The information provided in this dataset can be useful in future research studies as a baseline for calibration and validation of damage predictive models addressing the response of river bridges to the hydraulic loads associated with severe hydrodynamic impacts.
• Furthermore, it may be a useful testbed for other researchers, engineering consultants, GIS specialists and climate risk analysts seeking a well-documented case study for the application of frameworks relevant to transport network analysis and infrastructure risk.
• This dataset provides valuable information for researchers working in infrastructure flood resilience, consultants, and risk assessors, including the insurance sector, bridge owners and operators, as well as stakeholders working on the advancement of existing and new regulations for climate adaptation, design and management of mitigation actions.

## 1 Data Description

**Fig. 1:**
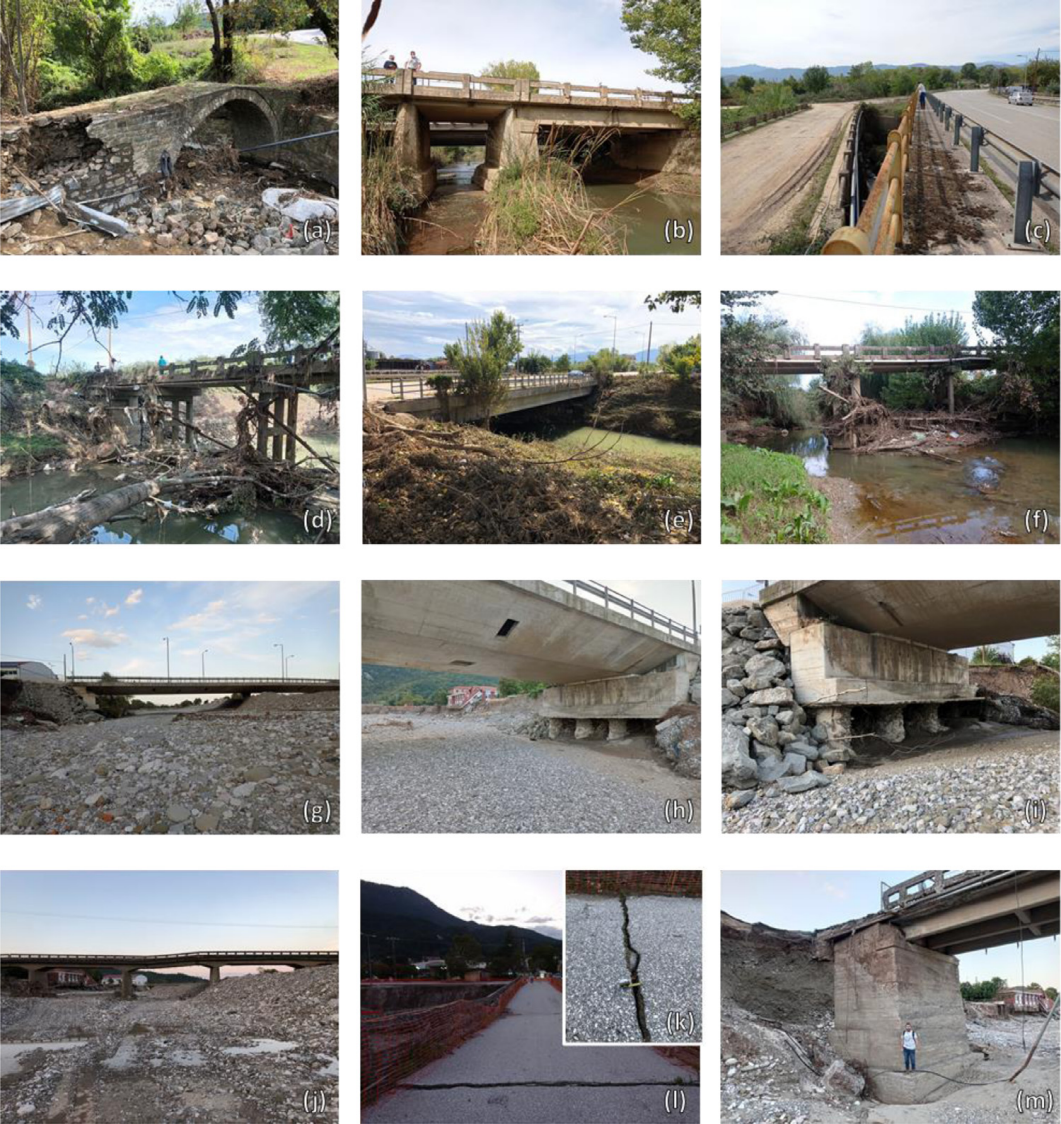

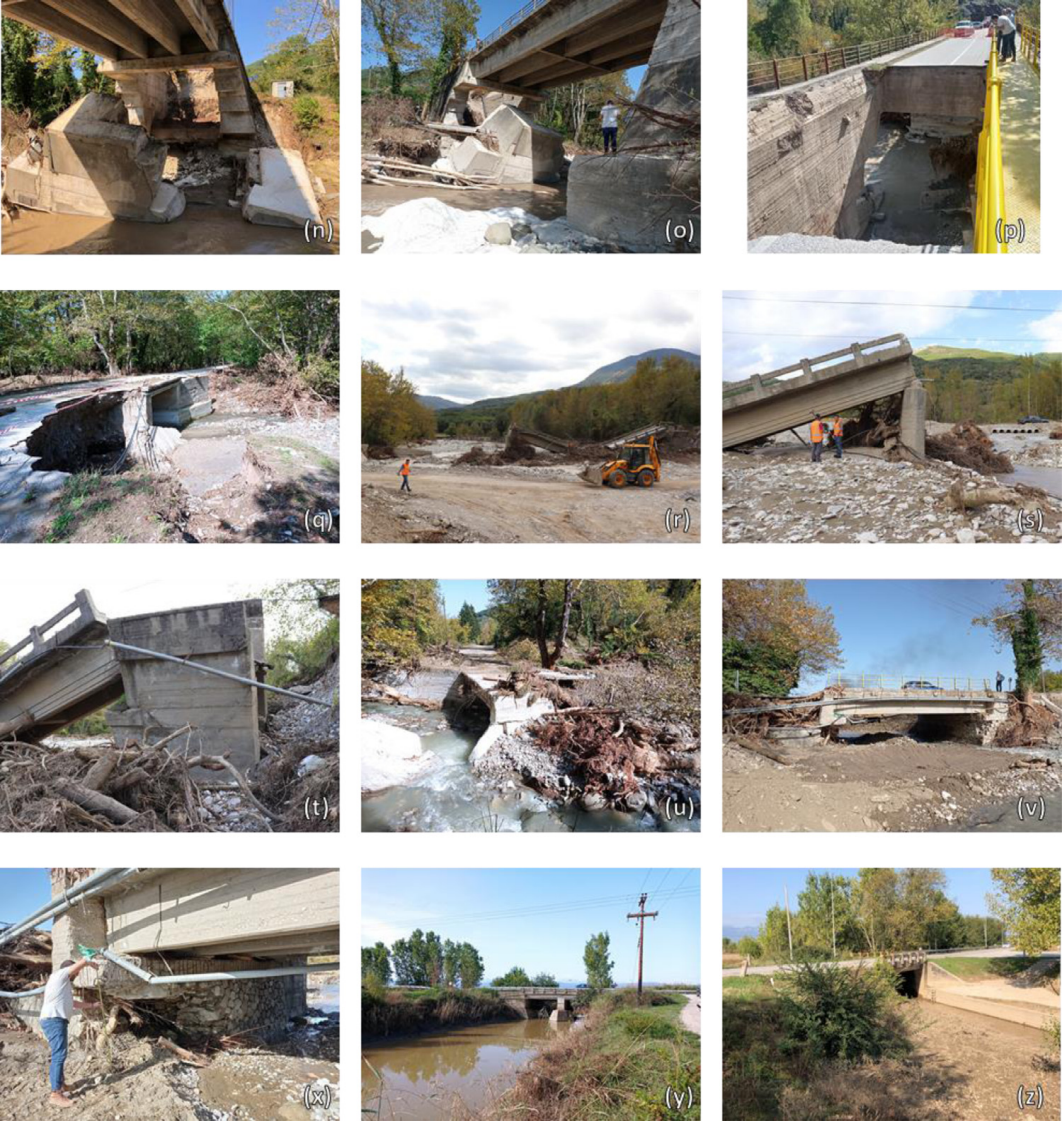
Photos of the inspected bridges: (a) No. 2; (b) No. 3; (c) No.3A on the left and No. 4 on the right; (d) No. 4; (e) No. 5; (f) No. 6; (g) – (i) No. 7; (j) – (m) No. 8; (n) – (p) No. 9; (q) No. 10; (r) – (t) No. 11; (u) No. 12; (v) – (x) No. 13; (y) No. 14; (z) No. 15.

The provided dataset includes an excel file (DATA_Karditsa_RiskAnalysis.xlsx) and a collection of images (Fig. 1). The former file includes inventory data for 117 bridges, serving the road and rail network in the city of Karditsa. Furthermore, it lists the main results from a damage reconnaissance study and the network-scale flood risk analysis carried out by Loli et al. (2022). Specifically, the list includes:

• A reference number preceded by a prefix, “BR” or “CU”, indicating bridge or culver, respectively.
• The observed damage, in terms of the damage level (see Loli et al., 2022), scales from 1 to 5, where 1 is assigned to minimal damage and 5 is assigned to complete failure. This damage assessment refers to on-site field investigations carried out after Medicane Ianos, from 3 – 15 October 2020. The field investigation focused on 15, out of the 117 bridges, for which a level of damage is provided. The NA value appears for the bridges that had not been inspected.
• X, Y coordinates in the 1987 Hellenic Geodetic Reference System (GGRS87).
• The construction time of the bridge is classified into 4 periods: before 1954, from 1955 – 1984, from 1985 – 1999, and after 2000.
• The type of transportation mode: rail or road
• The structural material: RC, metal, or masonry (making an indication of “bad” or “good” condition depending on whether there was evident corrosion or weathering of the material.
• Description of the static system: culvert, box-type, arch, truss bridge, isostatic/hyperstatic multi-frame.
• Basic geometric properties: Total length of the bridge (longitudinal), average height, number of piers, maximum support (pier/abutment) width, and the total width of wet supports (i.e. the sum of the pier and abutment widths that block the flow).
• The orientation relevant to the direction of the stream is denoted as favourable or oblique.
• The shape of the instream supports: circular, rectangular, or cutwater.
• The type of foundation: deep, surface, or unknown (when data is not available).
• Results from the flood risk analysis reported in Loli et al., 2022, listing:
• Estimate of the flood hazard (“low”, “medium”, or “high”) affecting each one of the bridge assets for the rainfall pattern produced by Medicane Ianos.
• Estimate of the bridge vulnerability to flood impacts (“low”, “medium”, or “high”).
• Prediction of the damage induced by the Medicane (in terms of the damage level, ranging from 1 – 5).
• Estimate of consequences of bridge failure for the network and its users (“very low”, “low”, “medium”, “high”, or “very high”).
• Estimate of flood risk (“very low”, “low”, “medium”, “high”, or “very high”).

**Fig. 2:**
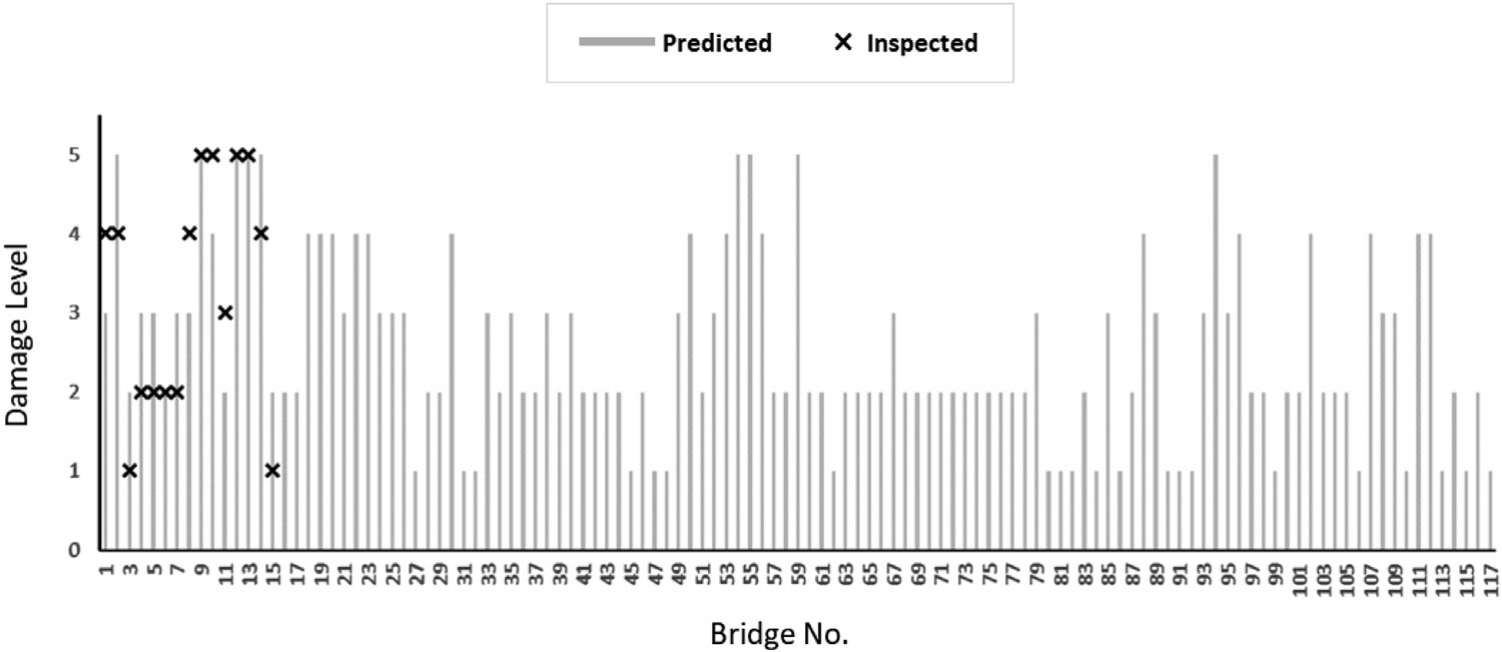
Predicted damage level for the entire bridge portfolio compared to observed damage level for the 15 bridges that were inspected after Medicane Ianos.

Fig. 2 plots the predicted damage level for each bridge (Loli et al, 2022) compared to the actual (inspected) damage level, for the bridges that were included in the reconnaissance study. The type and extent of damage (from minimal to severe) are visible in the images that are included in the dataset (and summarized in Fig. 1). Note that the image naming refers to the bridge reference number.

## 2 Experimental Design, Materials and Methods

The inventory dataset has been created based on the translation, organization and integration of survey reports archived by the Ministry of Environment and Energy of Greece (YPEKA). The surveys have been carried out in the framework of the Flood Hazard and Risk Maps produced by the Greek Government, following the 2007 Floods Directive of the European Commission [8]. The YPEKA has published (in Greek) one report for each one of the following rivers existing in the area: Enipeas, Farsaliotis, Sofaditis, Gavras, Kalentzis, Karampalis, Lipsimos, Makrirema, Mega Rema, and Pamisos. Material from these reports was collected, translated, and synthesized to create an inventory in excel (column “C” – column “P” in DATA_Karditsa_RiskAnalysis.xlsx).

A few days after Medicane Ianos struck Greece, on September 17-20 2020, causing severe and widespread damage to infrastructure [1], [2], [3],^5^,7], the US GEER Association deployed an interdisciplinary team of researchers and engineers to collect perishable field performance data from the affected regions. The mission made use of remote sensing technologies, employing optical and satellite imagery, in addition to conventional site characterization and structure inspections. The relevant report [9] provides a broad assessment of the medicane impact, based on a systematic collection of field observations and forensic engineering evidence.

A total of 15 bridges exhibiting diverse responses to flood impacts, from minimal damage to complete failure (Fig. 1), were inspected during 2 reconnaissance missions conducted in the prefecture of Karditsa (an area that suffered severe flooding). The first field deployment, using conventional site characterization and structure inspection tools, was carried out from 3 – 4 October 2020. The images of bridges included in this dataset (portrayed in Fig. 1) were taken by the lead author using a digital camera. A second deployment took place from 14 – 15 October, where UAV imagery was captured by a DJI Phantom 4 Professional (P4P) quadcopter to enable the construction of 3D digital models of the damaged bridges. The 2^nd^ mission (reported in [6]) focused on failed bridges of particular interest, namely No. 7, 8, 9 and 11. Oblique optical imagery was collected due to the complex geometry of the bridges, and additional aerial photos were captured manually, to eliminate “shadows” and ensure the creation of complete, 3D models. 60 ground control points were surveyed thoroughly covering the areas mapped, to ensure data quality verification and precise georeferencing of the collected data.

3D models of the bridges were created to enhance the observation of damage patterns, permitting the execution of measurements and numerical reproduction of the failure mechanisms (as in [5]). 3D visualizations of the digital models are freely available online, through the account of Professor D. Zekkos' Research Group @UC Berkeley on Sketchfab:

• Bridges No. 7 and No. 8: https://sketchfab.com/3d-models/mouzaki-bridges-53d168a58a164c7ba24ac50a5d888a6b
• Bridge No. 9: https://sketchfab.com/3d-models/mouzaki-karditsa-bridge-9-d54ac31279414dcb8a499414660ce0c1
• Bridge No 11: https://sketchfab.com/3d-models/mouzaki-bridge-4-d02a6fcb1f924faca9cd85015b6d29db

Based on field measurements and the interpretation of observations on the 3D digital models, the response of the inspected bridges was assessed in terms of the damage level (DL), on a scale from 1 – 5, where 1 implies no impact on the structural integrity and/or the functionality of the bridge and 5 is the ultimate failure state, associated with complete collapse or unrepairable damage. DL = 2 when minimal damage, such as minor spalling, and functional loss due to debris built-up was observed. DL = 3 in cases of moderate damage where repair works did not necessitate complete closure of the bridge. DL = 4 when damage is repairable yet demanding temporary closure of the bridge.

Column B in the DATA_Karditsa_RiskAnalysis.xlsx lists the observed (actual) DL of the inspected bridges after Medicane Ianos. This information was used to validate the effectiveness of a novel framework for the assessment of flood risk to bridges [4] in predicting flood-induced damage on bridges after Medicane Ianos. The predicted DL for the entire inventory (117 bridges) is listed in Column S of the same file (and plotted in Fig. 2). The risk assessment methodology is described in detail in the accompanying research article (Loli et al., 2022). It evaluates qualitatively, from very low to very high, (i) the hazard, associated with both scour and hydraulic actions; (ii) the vulnerability, a feature of the structure's characteristics; (iii) the consequences of bridge failure, an indicator of the bridge's importance; and (iv) the overall risk for the transport network. (i) – (iv) are included in the dataset for completeness and facilitation of future validation studies.

## Ethics Statements

Data collection and processing were performed following the relevant institutional and national regulations and legislation and the ethical guidelines of Data in Brief.

## CRediT authorship contribution statement

**Marianna Loli:** Conceptualization, Data curation, Formal analysis, Funding acquisition, Investigation, Methodology, Validation, Visualization, Writing – original draft. **George Kefalas:** Data curation, Formal analysis, Investigation, Methodology, Software, Visualization, Writing – review & editing. **Stavros Dafis:** Data curation, Formal analysis, Software, Visualization, Writing – review & editing. **Stergios Aristoteles Mitoulis:** Funding acquisition, Methodology, Project administration, Resources, Supervision, Writing – review & editing. **Franziska Schmidt:** Conceptualization, Methodology, Resources, Writing – review & editing.

## Declaration of Competing Interest

The authors declare that they have no known competing financial interests or personal relationships that could have appeared to influence the work reported in this paper.

## Data Availability

Flood Damage Inspection and Risk Indexing Data for an Inventory of Bridges in Central Greece (Original data) (Mendeley Data).
